# Proactive Coverage Area Decisions Based on Data Field for Drone Base Station Deployment

**DOI:** 10.3390/s18113917

**Published:** 2018-11-13

**Authors:** Bo Hu, Chuan’an Wang, Shanzhi Chen, Lei Wang, Hanzhang Yang

**Affiliations:** 1 State Key Laboratory of Networking and Switching Technology, Beijing University of Posts and Tele-communications, Beijing 100088, China; wangchuanan1010@126.com (C.W.); wlbupter@bupt.edu.cn (L.W.); yhz@bupt.edu.cn (H.Y.); 2 School of Information and Network Engineering, Anhui Science and Technology University, Chuzhou 233100, China; 3 State Key Laboratory of Wireless Mobile Communication, China Academy of Telecommunication Technology, Beijing 100191, China; chensz@datanggroup.cn

**Keywords:** drone base station, data field, proactive coverage areas, on-demand coverage radius

## Abstract

Using the drone base station (DBS) to alleviate the network coverage supply-demand mismatch is an attractive issue. Found in DBS-assisted cellular mobile networks, the deployment of DBSs to cope with the dynamic load requirements is an important problem. The authors propose a proactive DBS deployment method to enhance the DBS deployment flexibility based on network traffic. The proposed scheme uses potential value and minimum distance to decide the areas that most need to be covered, which are named as proactive coverage areas (PCAs), whereby the DBSs are assigned to cover those PCAs. Meanwhile, when the number of required DBSs is determined, the energy consumption is related to the coverage radius and the altitude of DBSs. Therefore, the proposed method further investigates the on-demand coverage radius and then obtains the altitude of DBSs. Simulations show that the proposed proactive DBS deployment method provides better coverage performance with a significant complexity reduction.

## 1. Introduction

Accompanying the development of the mobile Internet and Internet of Things (IoT), ubiquitous users and devices need access to a network, which is then followed by an increasing amount of traffic. Dense deployment of small base stations (BSs) is one intuitive way to handle the ever-increasing data traffic demands [1], however, the deployment strategy of current small BSs is in accordance with long-term network traffic behaviors, and lacks flexibility in location adjustment. Moreover, such a rigid access network is challenged in handling the difficult-to-predict traffic patterns caused by the temporal and spatial variations in user densities and user application rates [2]. Hence, to enhance network access flexibility for supporting massive dynamic connections and an uneven distribution of network traffic, the use of the drone base station (DBS) is an efficient approach for handling the traffic with better data rates in these heterogeneous scenarios [3,4].

The DBS, with the advantage of flexible deployment and low cost, can be deployed to help a ground network in providing high data rate coverage whenever there is an excessive service demand in space and time. Moreover, because a DBS is more robust against the change of environments compared to a static ground BS, it can be deployed to provide emergency communication connectivity in areas without infrastructure coverage, such as disaster scenes [4]. While there are several advantages for the use of DBSs, there are some issues that have not yet been addressed in the literature. To find a suitable number of DBSs along with their placement, in order to provide coverage to a set of user equipment (UE), is a very important question about the aerial wireless networks [5]. Therefore, at least two problems should be considered for deploying DBSs to cope with the dynamic load requirements.

- The coverage area of DBSs: One of the greatest challenges is to identify the proactive coverage area (PCA) that a DBS needs to cover. Especially in an overload condition caused by burst crowd traffic, the PCAs enclosed with more UE covered by the DBS benefits the network the most. Moreover, when the PCAs are determined, how to allocate DBSs to cover these areas is also one of the problems to be solved.
- The number of DBSs and the total energy consumption need to be considered. The authors assume that each DBS has a minimum and a maximum vertical altitude. Moreover, the energy consumption of a DBS is related to its altitude. Indeed, the higher the altitude, the larger the covered area, the higher the energy consumption. Thus, the on-demand coverage radius and the optimal altitude, as two key cost metrics, should be considered.

**Figure 1:**
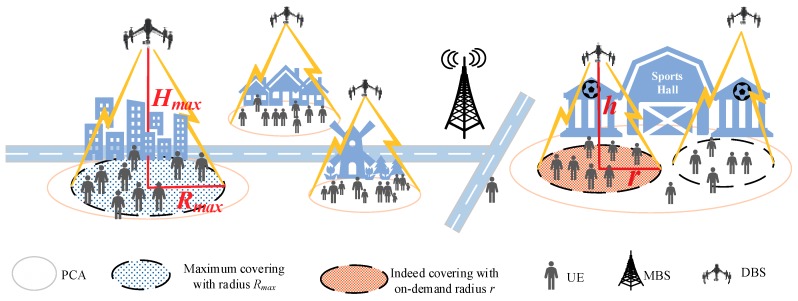
The deployment of multiple drone base stations (DBSs) in a drone-assisted wireless network. PCA: proactive coverage areas; UE: user equipment; MBS: macro base station.

The authors leverage the heterogeneity of the UE distribution to design algorithms for dynamically placing DBSs. Obtaining the spatial UE distribution and modeling it in the space and time domains plays an important role in characterizing and analyzing the performance of the network. The spatial UE distribution also has an impact on the network deployment. Especially in a drone-assisted wireless network, the placement of a DBS depends mainly on the distribution of UE [6]. Drawing inspiration from the theory of data field [7], an efficient multiple DBS deployment scheme is investigated. The proposed scheme uses potential value and minimum distance to decide the PCAs that most need to be covered, whereby the DBSs are assigned to cover those PCAs, as shown in Figure 1. Moreover, when the PCAs are determined, a “first-best-effort and second-patching” (FBE–SP) algorithm is designed to assign corresponding DBSs to cover them. Further, the authors considered two cost metrics to minimize the energy cost: the on-demand coverage radius and the optimal altitude. Generally speaking, the goal is to find the PCAs being covered by the DBSs to maximize the number of covered UE while ensuring the minimum energy cost.

The main contributions of this work can be concluded as follows:

- A novel method is proposed for deciding the PCAs. According to data field theory, a demand point with a larger potential value has more demand points gathered around it [8]. Then, the region centering on the demand point with local maximum potential value can be decided as a PCA. Compared to a heuristic DBS location decision, assigning DBSs to cover the decided PCAs has a lower complexity.
- To cover the decided PCAs by the supplied DBSs, treated as which DBSs serve which PCAs problem, the authors design the “first-best-effort and second-patching” (FBE–SP) algorithm to solve the problem.
- Meanwhile, the minimum energy cost mechanism is employed in this paper. The authors further consider the on-demand coverage radius and the optimal altitude as two cost metrics. The on-demand coverage radius is determined by the size of the area that the DBS actually needs to cover, while the corresponding optimal altitude can be more simply obtained by solving the linear equation between altitude and coverage radius.

The remainder of the paper is organized as follows. Section 2 discusses the proposed work in the context of related works. The system model and the problem formulation are presented in Section 3. Section 4 gives an efficient solution for the optimal deployment problem. Section 5 presents the simulation results. Finally, the work is concluded in Section 6.

## 2. Related Works

Recently, many researchers highlighted the important performance benefits achieved by the use of DBSs for wireless communication. Through field experiments, the authors in Reference [9] demonstrate the capability of DBSs for improving the signal strength in coverage holes when DBSs perform as the aerial relays of static ground BSs. Zhang et al. study the spectrum sharing of the DBS-assisted network modeled by the three-dimensional (3D) Poisson point process and find the optimal density of DBSs to maximize the network throughput while ensuring the network efficiency constraint [10]. Andreev et al. put forward a novel vision of moving access infrastructure to match dynamic user demand in a 5G network. They point out that the intelligent capable devices (drones or cars, for example) performing as moving access points will offer the operators an opportunity to dramatically boost system capacity [11]. To achieve fair performance among UE, Wu et al. maximize the minimum downlink throughput over all ground UE by optimizing the multi-user communication scheduling and association jointly with the DBS’s trajectory and power control [12]. These works are a significant effort to investigate a DBS’s potential to expand the coverage and capacity of existing ground wireless networks, but do not consider the optimal placement problem of a DBS. 

The optimal placement of a set of DBSs is a very challenging issue, which has been proven to be a NP-hard problem in most cases [13]. According to this problem, a set of waypoints is given by the macro BS (MBS) where DBSs can be placed, and entropy is used to provide the base for facility location [4]. In fact, the number of waypoints is difficult to specify in advance. Leveraging the air-to-ground (AtG) pathloss model in Reference [14], some researchers focus on the DBS deployment/placement optimization that maximizes specific performance metrics. Al–Hourani et al. provide an analytical approach to optimize the altitude of a DBS for providing maximum coverage for ground UE [15]. In contrast, by fixing the altitude, the horizontal locations of DBSs are optimized in Reference [16], and a bisection search is performed to find the minimum number of DBSs needed to provide full wireless coverage. As such, the altitude and the horizontal location of the DBS can be optimized jointly for different quality-of-service (QoS) requirements, which can be treated as a 3D placement optimization problem. The authors in Reference [17] highlight the properties of the DBS placement problem and study the 3D placement of a single DBS with the objective of maximizing the numbers of UE covered by the DBS. In reference [18], the 3D deployment problem of multiple DBSs has been formulated to maximize the user coverage while maintaining DBS to ground BS link qualities, and designed a per-drone iterated particle swarm optimization algorithm for solving the problem. Among these works, a heuristic algorithm is adopted mostly to select the location that maximizes specific performance to deploy DBSs. Thus, these approaches can be viewed as an optimal deployment problem from the network side.

Although the above proposals have addressed the problem of optimizing the deployment of DBSs, no other works have provided a comprehensive view of quickly deciding the PCAs and assigning the appropriate DBSs to cover them. The objective of this paper is an optimization problem formulated as a maximum coverage problem, while ensuring the minimum energy cost, which is recorded as the MCMC problem. To solve the MCMC problem, the data field theory is introduced, in which the demand point with local maximum potential values is selected as the coverage center point (CCP) of a PCA, ensuring the coverage maximization. Further, to minimize the energy cost, the on-demand coverage radius is determined by the size of the area that the DBS actually needs to cover, while the corresponding optimal altitude can be more simply obtained by solving the linear equation between altitude and coverage radius. The experimental results show that the current scheme is more suitable for operators to handle the difficult-to-predict traffic demand, especially in the environment involving large data sets.

## 3. System Model and Problem Formulation

Since the numbers of UE randomly distributed and deployed in the coverage area of a MBS is greater than the capacity of a MBS, a cost-efficient and high-unloading method must be devised. The authors limit the analysis to downlink, so DBSs are used to transmit data and the co-channel interference is ignored. Mobility is an important feature of a DBS, therefore an area does not need to be covered while there is few or no UE there. As UE moves, DBSs might follow them if needed, so here is the deployment of the DBSs for one snapshot of the UE positions.

### 3.1. System Model

A downlink wireless system is considered in this paper, where the MBS is used to provide generalized coverage, and a set of K={1, 2,…,K}. DBSs are deployed to relieve the overload caused by flash congested UE. When there is coverage overlap between the MBS and an active DBS, the authors assume that the DBS has the priority to serve the UE in the overlapped region once deployed. Placing a DBS with $R_{max}$ over a PCA obviously might cover as much UE as possible, but it also might cause a ring-shaped excess coverage area when the $R_{max}$ is greater than the radius of the PCA, as shown in Figure 1. Denoted by U={1, 2,…,M} the set of UE is to be offloaded in the coverage range of MBS, and their locations are given by $\{\left(x_{u},y_{u}\right);\;u=\left\{1,\;2,\ldots ,M\right\}$. Apparently, the minimum number of DBSs required to handle the congested UE is $K=\lceil \frac{N-U_{MBS}}{U_{DBS}}\rceil$, where ⌈·⌉ denotes the round up calculation and $U_{MBS}$ and $U_{DBS}$ account for the amount of UE that the MBS and a DBS can handle, respectively, while the 3D coordinates of the DBS can be denoted as $\left\{Z_{k}=\left(x_{k},y_{k},h_{k}\right);k=1,\;2,\ldots ,K\right\}$.

The authors assume that UE is in the coverage region of the DBS if the AtG link satisfies its guaranteed QoS requirement. A statistically generic AtG path loss model was studied in Reference [14]. The authors of Reference [19] refined the AtG path loss model and provided the useful mathematical formulae. The AtG communication links are mainly line-of-sight (LoS) and non line-of-sight (NLoS) links. Since fading is an essential factor in DBS-assisted networks, the authors consider Rician fading in LoS links, while using Rayleigh fading in NLoS links [20]. Considering a generic AtG path loss model, the received signal power of UE i from the DBS j is given by:

(1) $$\{\begin{array}{c} P_{LoS}\left(s_{u,k},h_{k}\right)=P_{0}d_{u,k}^{-\varsigma }\mu_{LoS}\\ P_{NLoS}\left(s_{u,k},h_{k}\right)=P_{0}d_{u,k}^{-\varsigma }\mu_{NLoS} \end{array}\;$$

where $P_{0}$ is the given transmit power of the DBS, $s_{u,k}$ is the horizontal distance between UE u and the DBS k, while $d_{u,k}$ is the spatial distance and $d_{u,k}=\sqrt{s_{u,k}^{2}+h_{k}^{2}}$, ς is the path loss exponent, $\mu_{LoS}$ and $\mu_{NLoS}$ are fading factors corresponding to the LoS and NLoS connection, respectively. The *NLoS* connection only has multiple reflection links, and $\mu_{NLoS}$ follows an exponential distribution with an average of ϖ. Compared with the NLoS connection, the *LoS* connection has not only a *LoS* link, but also multiple reflection links, which cause a Z-factor that should be considered.

The LoS probability of the connection between UE u and the DBS k is given by:

(2) $$p_{LoS}=\alpha {\left(180/\pi \cdot \arctan\left(h_{k}/s_{u,k}\right)-15\right)}^{\beta }\;$$

where α and β are constants which depend on the environment (suburban, urban, and ultra-dense urban, etc.). Correspondingly, the probability of NLoS is:

(3) $$p_{NLoS}=1-p_{LoS}.$$

Therefore, the average received power of UE u from the DBS k can be calculated by:

(4) $$\bar{P}\left(s_{u,k},\;h_{k}\right)=P_{LoS}\left(s_{u,k},h_{k}\right)p_{LoS}+P_{NLoS}\left(s_{u,k},h_{k}\right)p_{NLoS}.$$

**Figure 2:**
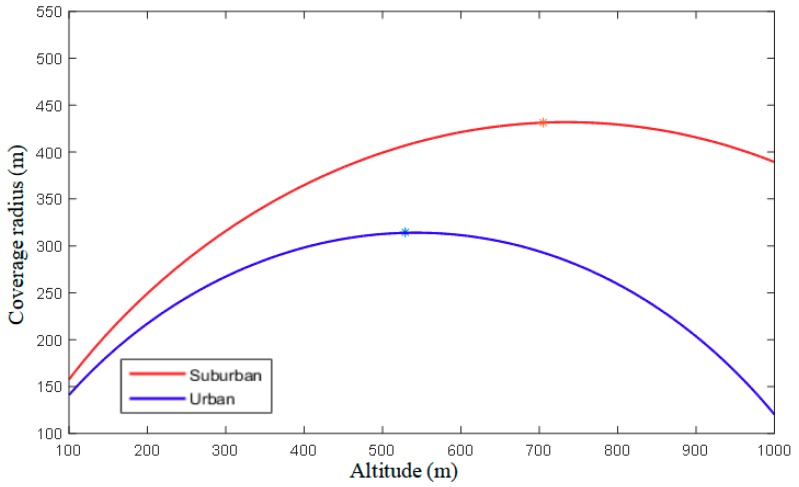
The relationship of the altitude and the coverage radius for a DBS.

The received power $\bar{P}\left(s_{u,k},h_{k}\right)$ is a function of distance $s_{u,k}$ and altitude $h_{k}$, meaning that, considering a generic AtG path loss model, the received power depends on horizontal distance and vertical altitude. To guarantee QoS, it needs to guarantee that the received power $\bar{P}\left(s_{u,k},h_{k}\right)$ must exceed a certain given threshold $P_{min}$ corresponding to the QoS requirement. According to Equation (4), for a given $P_{min}=-70$ dBm, the relationship between the altitude and the coverage radius for a DBS can be obtained, as shown in Figure 2.

Figure 2 shows, by increasing the altitude of a DBS, the coverage radius first increases and then decreases. That is because, when in higher altitudes, the increased LoS probability is higher than the NLoS probability and, in turn, the coverage radius increases. Conversely, the received power also is dependent on the spatial distance between the UE and the DBS, so, after the altitude exceeds a specific height, the coverage radius decreases. When the altitude reaches the specific value $h^{*}$, the maximum coverage radius $R_{max}$ is obtained, as the extreme point $\left(h^{*},\;R_{max}\right)$ shown in Figure 2.

### 3.2. Problem Formulation

Deploying the DBSs with $R_{max}$ over every PCA obviously might cover as much UE as possible, but it also might cause a ring-shaped excess coverage area, as shown in Figure 1. Furthermore, when multiple DBSs cover the same PCA, more overlap is created. Therefore, the on-demand coverage radius is considered. Conversely, the DBS’s energy consumption is related to the coverage radius and the altitude [21]. To minimize cost, it is essential to find an optimal altitude $h_{opt}$ that minimizes the cost while ensuring the service of the covered UE. The objective is treated as a maximum coverage and minimum cost (MCMC) optimization problem, as previously noted.

Denoted by $A_{l}$ the set of UE enclosed in the PCA l and $\left|A_{l}\right|$ is the cardinality of the set $A_{l}$, and the objective function is to maximize the total amount of coverage for UE in all the PCAs:

(5) $$\max\overset{L}{\underset{l}{\sum }}\left|A_{l}\right|$$

$$\;\begin{array}{cc} \mathrm{subject}\;\mathrm{to} & \left(1\right)L\leq K\\ & \left(2\right)s_{u,k}\leq R_{max}\;\forall u\in A_{l},\;\forall k\in K\\ & \left(3\right)h_{k}\leq H_{max}\;\forall k\in K\\ & \left(4\right)\bar{P}\left(s_{u,k},\;h_{k}\right)\geq P_{min}\;\forall u\in A_{l},\;\forall k\in K\; \end{array}$$

where *L* is the number of PCAs, $H_{max}$ is the maximum altitude of the DBS, and the constraints in Equation (5): (1) indicates that the number of PCAs is no more than the number of supplied DBSs; (2) is the horizontal distance constraint, which means that the distance from any UE u in PCA *l* to the corresponding DBS k must be less than $R_{max}$; (3) denotes that the altitude of each DBS should be less than the given $H_{max}$; (4) denotes that the received power $\bar{P}\left(s_{u,k},\;h_{k}\right)$ must exceed the given threshold $P_{min}$.

Regarding a given *K* DBS being deployed, it is not easy to find the optimal 3D locations that satisfy the objective function of Equation (5). First, determine *L* horizontal coverage areas for DBS deployments and then optimize the flight altitude for minimizing the cost, which is a NP-Hard combinatorial optimization problem, which is very complex to find the optimal solution by solving the mathematical model. One way to reduce the computational complexity and still reach a feasible solution is to split the target task into different domains. Thus, a low complexity algorithm is proposed to solve the MCMC problem.

## 4. Efficient Solution for the MCMC Problem

Finding PCAs being covered by the supplied DBSs that maximize the numbers of covered UE is a very complicated optimization problem. Adding two new dimensions to the problem, which are the altitude and on-demand coverage radius of the DBS, makes the MCMC problem even more complex. Hence, to solve the MCMC problem, the idea is to divide the problem into three sub-problems: deciding PCAs, duly placing-and-operating the DBSs, and optimal 3D location decisions. The solution to the first sub-problem is to determine the PCAs enclosed with maximum UE, while the second is to assign the DBSs to the PCAs, and the last is to find the optimal 3D location of each DBS.

### 4.1. Deciding Proactive Coverage Areas

Within the coverage range of MBS, each item of UE i is considered a data demand point $f_{i}$, where $f_{i}=\left(x_{i},y_{i}\right)$, and the $x_{i}$ and $y_{i}$ are the coordinates of the UE i in the X, Y axes. Thus, the physical field is extended to a data field. Moreover, a potential function is given for calculating the potential value of any point in data space, which is used to describe quantitatively the effect of each point on other points in the field. Denoted by $F=\left\{f_{1},f_{2},\ldots ,f_{n}\right\}$ a data set made of n demand points, the potential value of demand point $f_{i}$ can be expressed as:

(6) $$\psi_{i}=\sum_{j=1}^{n}m_{j}e^{-{\left(\frac{s_{i,j}}{\sigma }\right)}^{\eta n}}\;$$

where $m_{j}$ is the mass of demand point $f_{i}$, σ is an impact factor, and $s_{i,j}$ is the distance between $f_{i}$ and $f_{j}$, while η is the distance index. Please note that the mass of each demand point must satisfy $m_{j}\geq 0$ and $\sum_{j=1}^{n}m_{j}=1$.

Reference [22] has proven that the spatial distribution of the data field mainly depends on impact factor σ and is insensitive to the value of η. When set η=2, a Gaussian potential function which has a favorable mathematical property is obtained. Thus, η=2 is fixed. The impact factor σ has an impact on the final potential distribution. This paper will optimize σ using the potential entropy method. The potential entropy E is given by:

(7) $$E=-\sum_{i=1}^{n}\frac{\psi_{i}\log\left(\frac{\psi_{i}}{Z}\right)}{Z}\;$$

where $Z=\sum_{i=1}^{n}\psi_{i}$ is a normalization factor.

The potential entropy E is used to measure the uncertainty of potential field distribution. When the potential value of one demand point is equal to that of another demand point, then the uncertainty of the potential field distribution is the largest, which corresponds to the maximum potential entropy. Otherwise, minimum potential entropy is generated if the potential values are unevenly distributed. Therefore, the optimal impact factor σ could be obtained by the minimum potential entropy E.

The $\delta_{i}$ is the minimum distance between the point $f_{i}$ and any other point with higher potential value, which can be calculated as follows:

(8) $$\delta_{i}=\underset{j:\psi_{j}>\psi_{i}}{\min}\left(s_{i,j}\right).$$

Regarding the demand point with the highest density, the authors conventionally take $\max_{j}\left(s_{i,j}\right)$ as $\delta_{i}$.

According to data field theory, the potential value of a demand point stands for the aggregate degree of its surrounding demand points. The larger its potential value, the more demand points are gathered around it. As anticipated, CCP is usually the demand point with local maximum potential value. Thus, the demand point $f_{i}$, with relatively high $\psi_{i}$ and large $\delta_{i}$, can be regarded as a CCP. The authors identify a CCP by computing the thresholds of the potential value and distance. To avoid any two CCPs being in an area with radius $R_{max}$, the authors set $\delta_{th}>R_{max}$, where $\delta_{th}$ is the threshold of distance $\delta_{i}$, while the threshold $\psi_{th}$ of potential value $\psi_{i}$ is determined by searching an ‘elbow point’ according to the method presented in Reference [23]. Further, the area with a certain radius, and centering on a CCP, can be considered as a PCA. The PCA decision algorithm proposed is able to spot PCAs of any size and shape, therefore, maximum coverage performance can be achieved if such PCAs are duly served. The PCA decision algorithm is detailed in Algorithm 1.

**Algorithm 1.** PCAs decision algorithm for DBSs placement
1:Obtain the impact factor σ that minimizes the entropy potential E, and calculate the potential value $\psi_{i}$ for each demand point $f_{i}$ using Equation (6).2:Use Equation (8) to calculate the minimum distance $\delta_{i}$ for each demand point $f_{i}$.3:Obtain the threshold values, $\psi_{th},\delta_{th},\psi_{min}$.4:Exclude the noisy demand points the demand points i characterized by $\psi_{i}<\psi_{min}$.5:Identify the coverage center points (CCPs) of all those demand points $f_{i}$ characterized by $\psi_{i}>\psi_{min}$ and $\delta_{i}>\delta_{th}$.6:Assign each remaining demand point to the nearest CCP characterized by a higher potential value and form the clusters.7:Decide PCAs: The area centering on the CCP and enclosing all the members of the i-th cluster is decided as a PCA. Obtain the finalized PCAs.


### 4.2. Placing-And-Operating the DBSs

Once the PCAs are decided, the next task is to determine which DBSs serve which PCAs, which is treated as an on-demand assign problem. Conceivably, how to place the supplied DBSs to cover the decided PCAs can significantly affect the coverage performance, which is one of the main performance metrics that the authors aim to improve. The authors do not consider the distance between the DBS and the target PCA to be covered, for simplicity.

Next, the authors describe the “first-best-effort and second-patching” algorithm (FBE–SP) for solving the above on-demand assign problem in detail. 

First-Best-Effort: The authors divide the numbers of congested UE in a PCA by the service capability of a DBS; thus, the quotient and remainder are outputted. The quotient is the number of DBSs for first-best-effort covering the PCA to offload the congested UE.

Second-Patching: The remaining available DBSs will share a certain number of remainders for patching coverage. Then follows Algorithm 2 (FBE–SP).

Following the FBE–SP process, the number of DBSs required for covering each PCA can be obtained.

(9) $$C_{j}=\{\begin{array}{cc} q_{j}+1 & \mathrm{if}\;\mathrm{the}\;j\mathrm{th}\;\mathrm{PCA}\;\mathrm{is}\;\mathrm{executed}\;\mathrm{second}\;\mathrm{patching}\\ q_{j} & \mathrm{otherwise} \end{array}$$

where $C_{j}$ is the number of DBSs assigned to cover the j-th PCA.

**Figure 3:**
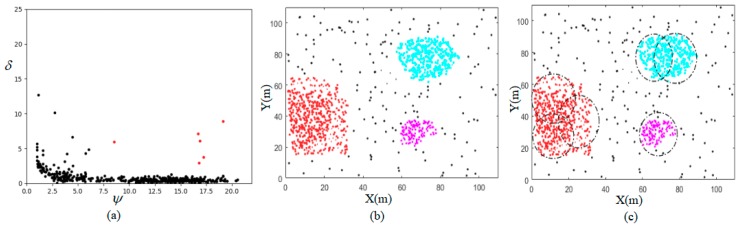
The visual example of the proposed algorithm phases: (**a**) Decision graph phase, (**b**) Deciding PCAs phase and (**c**) Placing-and-Operating the DBSs phase.

The visual example of the proposed algorithm phases is shown in Figure 3. According to the decision graph in Figure 3a, three demand points are selected as CCPs by the threshold method and each CCP has a high potential value ψ and large distance δ. To form PCAs, the demand points are assigned to the nearest CCP characterized by a higher potential value. Figure 3b shows three areas centered on each CCP are selected as PCAs, and these PCAs are marked in color. The FBE–SP determines the number of required DBSs for covering each PCA, and assigns these DBSs to cover the corresponding PCA, as shown in Figure 3c.

**Algorithm 2.** First-Best-Effort and Second-Patching algorithm (FBE–SP)**Initialization:** Set the number of supplied DBSs as *K*, and the corresponding capacity is *U_DBS_* of each DBS, a set of congested UE of PCA, A = {*a*_1_, *a*_2_, …, *a_L_*}, where *a_j_* represents the numbers of UE in the j-th PCA, |.| denotes the cardinal number. 
1:Regarding each PCA j, calculate $\left(q_{j},p_{j}\right)=\lfloor a_{j}/U_{DBS}\rfloor$ where ⌊·⌋ denotes the round down calculation, and $q_{j}$, $p_{j}$ denote the quotient and remainder, respectively.2:Assign $q_{j}$ DBSs to PCA j for first-best-effort covering.3:Obtain the number of the first required DBSs, $K_{1}=\sum_{j=1}^{\left|A\right|}q_{j}$.4:When *K > K*_1_, sort the |A| remainders in descending order and select the top *K* − *K*_1_ remainders. Each remainder corresponds to a Second–Patching PCA.5:Assign the remaining *K* − *K*_1_ DBSs to cover the Second–Patching PCAs.


### 4.3. 3D Location Optimizing for Energy Efficiency

Subsequently, the authors give a mechanism to optimize the 3D location of a DBS. The authors decouple the 3D placement problem in the horizontal and vertical dimensions for simplicity. Since the coverage area of a DBS is a circular disc, placing the circular disc on the horizontal plane corresponds to placing the DBS horizontally. Further, placing a DBS centering on a CCP ensures that the summation of the distance to all the UE covered by the DBS is kept to a minimum. Thus, the coordinates of the CCP can be seen as the horizontal location of the DBS. Concurrently, the optimal altitude is the vertical dimension. The procedure of which can be summarized as the following.

Use Algorithm 1 to analyze the UE distribution information currently calculated by the data field. As a result, the actual PCAs needed to be covered by the DBSs can be obtained, assuming the number of PCAs is *J*. The FBE–SP algorithm determines the number of DBSs required to cover each PCA. Given $C_{j}$ DBSs for covering the j-th PCA, should decide the locations $\left\{Z_{j}^{n},n=1,2,\ldots C_{j}\right\}$ of these DBSs, where $Z_{j}^{n}=\left(x_{j}^{n},\;y_{j}^{n},h_{j}^{n}\right)$.

To discover the horizontal locations of the $C_{j}$ DBSs, divide the j-th PCA into $C_{j}$ sub-PCAs, and each sub-PCA is covered by a DBS. Use Equation (6) to calculate the potential value for each item of UE in sub-PCA and select the UE with a local maximum potential value as the CCP of each sub-PCA. The locations of these CCPs are denoted by $\left\{\Gamma_{j}^{n},n=1,2,\ldots C_{j}\right\}$, where $\Gamma_{j}^{n}=(s_{j}^{n},t_{j}^{n})$ denotes the X, Y axes of the CCP. Thus, the DBS horizontal location $\left(x_{j}^{n},y_{j}^{n}\right)$ over the n-th sub-PCA can be calculated as:

(10) $$\left(x_{j}^{n},y_{j}^{n}\right)=(s_{j}^{n},t_{j}^{n}).$$

Next, decide the optimal altitude of DBS. Reference [19] proves that there is a linear relationship between the altitude and horizontal coverage radius. Then, determine the coverage radius of DBS. Calculate the distance $\lambda_{j}^{n}$ between the CCP $\Gamma_{j}^{n}$ and the farthest UE in n-th sub-PCA.

(11) $$\lambda_{j}^{n}=\underset{f_{j}^{n}\epsilon D_{j}^{n}}{\max}\left(d_{\Gamma_{j}^{n},f_{j}^{n}}\right)\;$$

where $D_{j}^{m}$ denotes the UE set enclosed in the n-th sub-PCA.

To reduce the ring-shaped excess coverage area caused by deploying the DBS with $R_{max}$, the on-demand coverage radius is needed. The on-demand coverage radius $r_{j}^{n}$ is determined by the size of the area that the DBS actually needs to cover.

(12) $$r_{j}^{n}=\{\begin{array}{cc} \lambda_{j}^{n} & \mathrm{if}\;\mathrm{the}\;\mathrm{distance}\;\lambda_{j}^{n}\;\mathrm{is}\;\mathrm{less}\;\mathrm{than}\;R_{max}\\ R_{max} & \mathrm{otherwise} \end{array}\;$$

Considering Equation (3), the received power is related to the altitude and coverage radius of the DBS. Mathematically speaking, $\bar{P}\left(r_{j}^{n},\;h_{j}^{n}\right)$ only depends on $h_{j}^{n}$ and $r_{j}^{n}$. Thus, for a given $r_{j}^{n}$, the optimal altitude problem can be formulated as:

(13) $$\underset{h_{j}^{n}}{\max}\bar{P}\left(r_{j}^{n},\;h_{j}^{n}\right)$$

$$s.t.\;\bar{P}\left(r_{j}^{n},\;h_{j}^{n}\right)\geq P_{min}.$$

Let $h_{j}^{*n}$ be the optimal DBS altitude and the $h_{j}^{*n}$ can be obtained by solving the problem (13). As evidenced in Reference [19], the optimal altitude is proportional to coverage radius. Therefore, when the on-demand coverage radius $r_{j}^{n}$ of DBS is decided, the problem (13) can be solved.

### 4.4. Complexity Analysis

The complexity of the proposed scheme is analyzed for further comparison. The authors mainly consider the influence of computational complexity in placing and operating DBSs. First, the authors compare the computational complexity of finding PCAs. In order to decide PCAs, the main computational operations are to calculate potential value and the minimum distance, as in Equations (6) and (8), respectively. Thus, the complexity of deciding PCAs is $O\left(M^{2}\right)$, even in the worst case. Once the PCAs are decided, the main complexity depends on determining which DBSs serve which PCAs, as described in Algorithm 2. Considering the FBE–SP algorithm, the main computational operations are to calculate the numbers of UE in each PCA, and the complexity is O(*M* *× L*), where *L* is the number of PCAs. Another main computational operation depends on obtaining the optimal altitude by solving the maximum problem, as in Equation (13). Since the problem (13) is a linear problem, the complexity is *O*(1). Based on the above analysis, the overall complexity of the proposed scheme is $O\left(M^{2}\right)$ + O(K×M) + O(1). Since the number of PCAs *L* is far less than the numbers of UE *M*, the overall complexity is approximately $O\left(M^{2}\right)$.

## 5. Simulation Analysis

The authors test the proposed scheme in urban and suburban environments. According to the methods used by Reference [24], the AtG propagation parameters of the two environments are shown in Table 1.

### 5.1. Simulation Implementation

The authors use MATLAB software as a simulation platform. During the simulation, the authors consider a 2 × 2 km area, in which 1000 items of UE are distributed in a number of different ways. As a statistical parameter related to the distribution of UE, the coefficient of variation (CoV) is first proposed in Reference [25]. The current authors use the value of CoV as the index of UE aggregation. CoV=1 indicates that the UE is distributed uniformly in the area, while CoV>1 indicates the aggregation of UE located around hotspots [19]. The simulation has the following numerical parameter settings: number of DBSs K=6, the service capability of a DBS $U_{\mathrm{DBS}}=200$ UE, the transmit power $P_{0}=30$ dBm, and the given threshold $P_{min}=‒70$ dBm.

**Figure 4:**
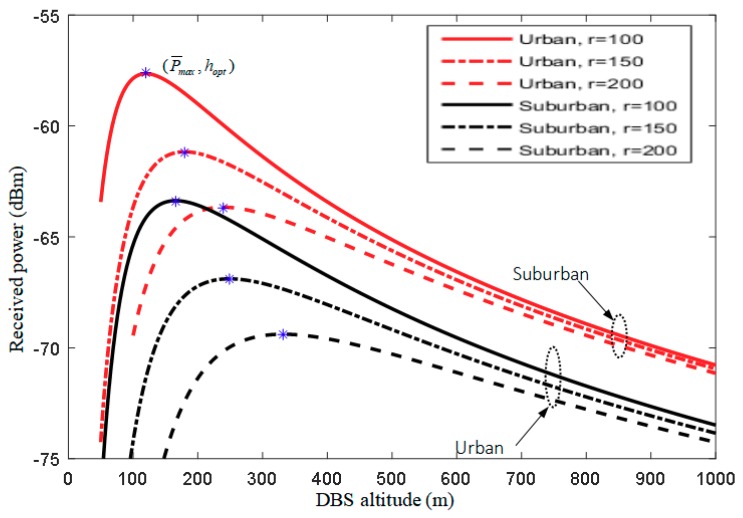
Received power vs. optimal DBS altitude under different on-demand coverage radius.

Figure 4 illustrates the received power versus DBS altitude for suburban and urban environments. It is noticed that on each performance curve exists a crest point, where the X and Y axes of the point represent maximum received power of the UE and the optimal DBS altitude, respectively. Furthermore, the optimal DBS altitude $h_{opt}$ increases as the on-demand coverage radius *r* increases, while the maximum received power ${\bar{P}}_{max}$ is reversed. Note that the optimal DBS altitude $h_{opt}$ is proportional to the given *r*, and the slope is determined by the environment. Therefore, once the on-demand coverage radius *r* of the DBS is decided, the corresponding $h_{opt}$ can be obtained more simply by solving the linear equation.

**Figure 5:**
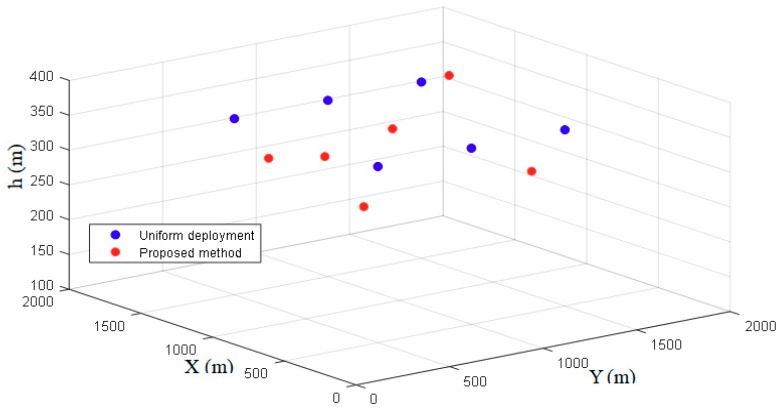
3D locations of DBSs.

The authors compare the proposed scheme with the uniform deployment scheme proposed in Reference [20]. The uniform deployment is a greedy heuristic scheme, which randomly selects a location to place a DBS with maximum coverage radius and removes the covered region from consideration when placing the next DBS. Figure 5 shows the 3D placement of the DBSs of the two schemes in an urban environment. It can be seen that using the proposed scheme, the DBSs are placed to serve maximum numbers of UE with on-demand coverage radius and dynamically adjust the altitude to guarantee the QoS requirement. Therefore, the horizontal location of each DBS is corresponding to the coordinate of the CCP and the vertical coordinate is the optimal altitude $h_{opt}$. Using the uniform deployment scheme, the DBSs are distributed almost evenly on the horizontal plane, while in the vertical dimension they have fixed altitude.

### 5.2. Performance Evaluation

In the following experiments, we evaluate the performance of the proposed scheme using three indicators including the covered ratio, delay time, and average transmit power. Note that the delay time considered for the mapping is included as the processing delay in the general deployment governing of the network. In order to measure the performance in DBS deployment based on the proposed scheme, the performance is evaluated against with the uniform deployment pattern.

**Figure 6:**
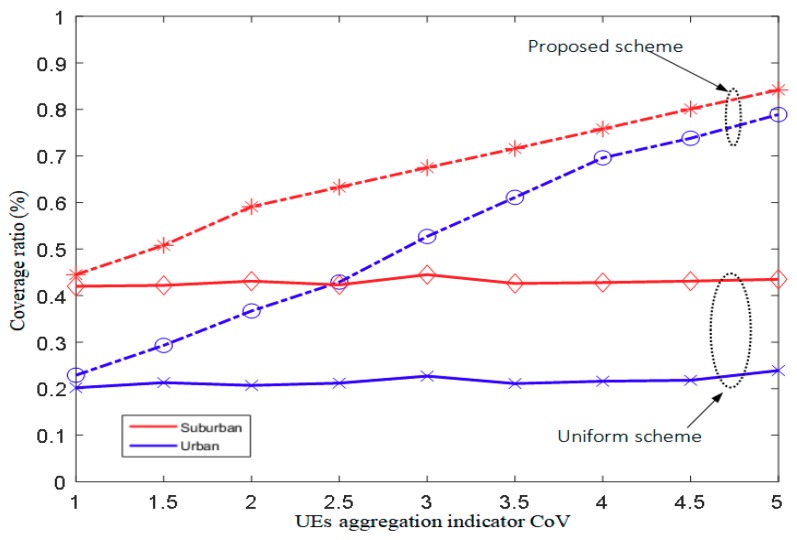
Coverage ratio of covered UE vs. coefficient of variation (CoV).

The coverage ratio of covered UE versus the CoV is depicted in Figure 6. As seen in this figure, more UE is covered in the suburban environment than in the urban environment because the former has a larger coverage radius of the DBS. Moreover, for the proposed scheme, the coverage ratio increases as the CoV increases and performs better than the uniform deployment scheme over the entire range of the CoV.

**Figure 7:**
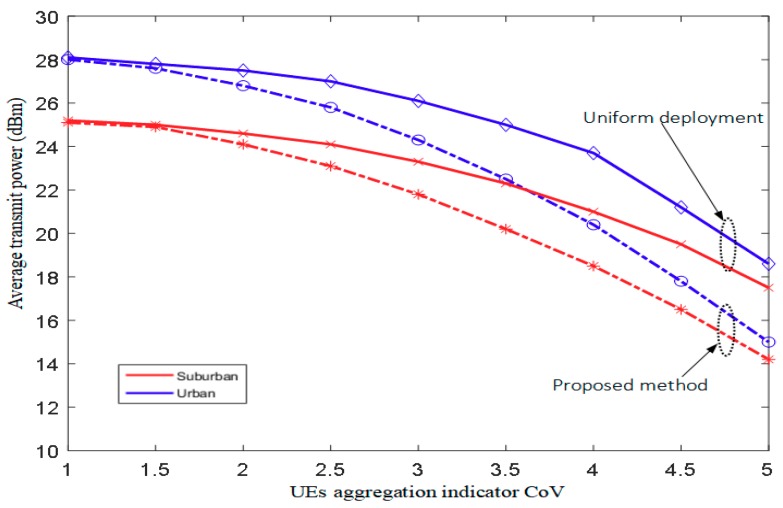
Average transmit power vs. CoV.

Figure 7 illustrates the average transmit power versus the CoV for urban and suburban environments. When CoV=5, compared with the uniform deployment scheme, the average transmit power of the proposed scheme has significant reductions of 4.4 and 2.5 dBm for urban and suburban environments, respectively. Moreover, with the increasing CoV, which means the higher aggregation of UE located around PCAs, the average transmit power decreases for the two schemes.

**Figure 8:**
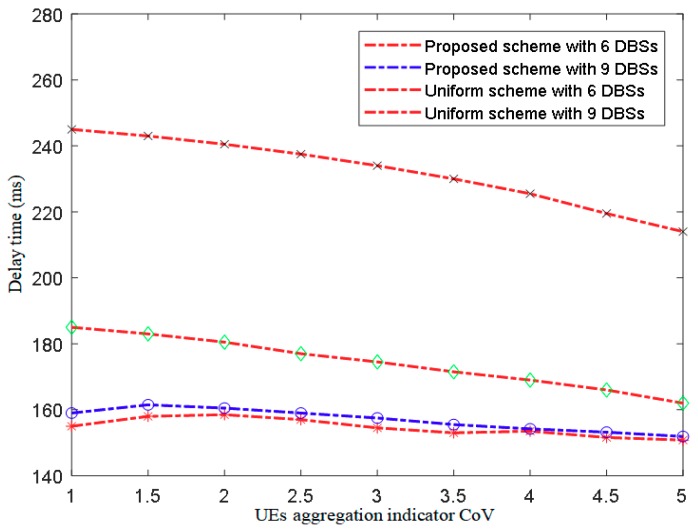
Delay time vs. CoV.

The environmental impact is not compared for delay time, mainly refers to processing time, and is not able to authenticate results of delay time. The plot in Figure 8 shows the comparison of the delay time observed in DBS deployment.

From Figure 8, it can be seen that the overall deployment governing delay of the proposed scheme is less than the uniform scheme. Furthermore, the delay time for the uniform scheme varied with an increase in DBSs since more iterations are required to deploy the additional DBSs. However, for the proposed scheme, the overall positioning delay is mainly related to the density of UE, and the number of DBSs has little influence on it.

## 6. Conclusions

A multiple DBS placement scheme is investigated in this paper. The proposed model uses potential value and minimum distance to decide the PCAs, whereby DBSs are assigned to cover the PCAs based on an FBE–SP algorithm. Moreover, to minimize the energy cost, the on-demand coverage radius and optimal altitude are considered. Due to the linear relationship between the coverage radius and altitude of DBS, the corresponding altitude can be obtained simply when the on-demand coverage radius is determined. The proposed scheme is compared favorably against a well-known benchmark scheme in the coverage performance.

Additionally, the proposed scheme has a lower complexity and higher operability, and is very suitable for operators to handle the difficult-to-predict traffic demand quickly by dynamically deploying DBSs and effective DBS migration strategies.

## Figures and Tables

**Table 1:** Air-to-ground (AtG) propagation parameters.

	Urban	Suburban
$\left(\mu_{LoS},\mu_{NLoS}\right)$	(1,20)	(0.1,21)
(a,β)	(9.61,0.16)	(4.88,0.43)

## References

[1] Kela P., Turkka J., Costa M. (2015). Borderless mobility in 5G outdoor ultra-dense networks. IEEE Access.

[2] Zhang N., Zhang S., Yang P., Alhussein O., Zhuang W., Shen X. (2017). Software defined space-air-ground integrated vehicular networks: Challenges and solutions. IEEE Commun. Mag..

[3] Chandrasekharan S., Gomez K., Al-Hourani A., Kandeepan S. (2016). Designing and implementing future aerial communication networks. IEEE Commun. Mag..

[4] Sharma V., Sabatini R., Ramasamy S. (2016). UAVs Assisted Delay Optimization in Heterogeneous Wireless Networks. IEEE Commun. Lett..

[5] Kalantari E., Yanikomeroglu H., Yongacoglu A. On the number and 3D placement of drone base stations in wireless cellular networks. Proceedings of the Vehicular Technology Conference.

[6] Gupta L., Jain R., Vaszkun G. (2016). Survey of important issues in UAV communication networks. IEEE Commun. Surv. Tutor..

[7] Iellamo S., Lehtomaki J.J., Khan Z. Placement of 5G Drone Base Stations by Data Field Clustering. Proceedings of the IEEE 85th Vehicular Technology Conference.

[8] Rodriguez A., Laio A. (2014). Clustering by fast search and find of density peaks. Science.

[9] Miranda K., Molinaro A., Razafindralambo T. (2016). A survey on rapidly deployable solutions for post-disaster networks. IEEE Commun. Mag..

[10] Zhang C., Zhang W. (2017). Spectrum sharing for drone networks. IEEE J. Sel. Areas Commun..

[11] Andreev S., Petrov V., Dohler M., Yanikomeroglu H. (2017). Future of Ultra-Dense Networks Beyond 5G: Harnessing Heterogeneous Moving Cells. arxiv.

[12] Wu Q., Zeng Y., Zhang R. (2018). Joint trajectory and communication design for multi-UAV enabled wireless networks. IEEE Trans. Wirel. Commun..

[13] Zorbas D., Pugliese L.D.P., Razafindralambo T., Guerriero F. (2016). Optimal drone placement and cost-efficient target coverage. J. Netw. Comput. Appl..

[14] Al-Hourani A., Kandeepan S., Jamalipour A. Modeling air-to-ground path loss for low altitude platforms in urban environments. Proceedings of the IEEE Global Communications Conference.

[15] Al-Hourani A., Kandeepan S., Lardner S. (2014). Optimal LAP altitude for maximum coverage. IEEE Wirel. Commun. Lett..

[16] Lyu J., Zeng Y., Zhang R., Lim T.J. (2017). Placement optimization of UAV-mounted mobile base stations. IEEE Commun. Lett..

[17] Bor-Yaliniz R.I., El-Keyi A., Yanikomeroglu H. Efficient 3-D placement of an aerial base station in next generation cellular networks. Proceedings of the IEEE International Conference on Communications.

[18] Shi W., Li J., Xu W., Zhou H., Zhang N., Zhang S., Shen X. (2018). Multiple drone-cell deployment analyses and optimization in drone assisted radio access networks. IEEE Access.

[19] Alzenad M., El-keyi A., Lagum F., Yanikomeroglu H. (2017). 3D placement of an unmanned aerial vehicle base station (UAV-BS) for energy-efficient maximal coverage. IEEE Wirel. Commun. Lett..

[20] Xie J., Dong C., Wang H., Li A. Performance analysis of drone small cells under inter-cell interference. Proceedings of the Ninth International Conference on Wireless Communications and Signal Processing.

[21] Mozaffari M., Saad W., Bennis M., Debbah M. (2016). Efficient deployment of multiple unmanned aerial vehicles for optimal wireless coverage. IEEE Commun. Lett..

[22] Li D., Wang S., Gan W., Li D. (2011). Data field for hierarchical clustering. Int. J. Data Warehous. Min..

[23] Zhao P., Qin K., Ye X., Wang Y., Chen Y. (2016). A trajectory clustering approach based on decision graph and data field for detecting hotspots. Int. J. Geogr. Inf..

[24] Yang P., Cao X., Yin C., Xiao Z., Xi X., Wu D. (2017). Proactive drone cell deployment: Overload relief for a cellular network under flash crowd traffic. IEEE Trans. Intell. Transp. Syst..

[25] Mirahsan M., Schoenen R., Yanikomeroglu H. (2015). HetHetNets: Heterogeneous traffic distribution in heterogeneous wireless cellular networks. IEEE J. Sel. Areas Commun..

